# New Trends and Therapies for Familial Hypercholesterolemia

**DOI:** 10.3390/jcm11226638

**Published:** 2022-11-09

**Authors:** Fahad Alnouri, Raul D. Santos

**Affiliations:** 1 Cardiovascular Prevention Unit, Department of Adult Cardiology, Prince Sultan Cardiac Centre, Riyadh 12233, Saudi Arabia; 2 Lipid Clinic Heart Institute (InCor), Medical School Hospital, University of Sao Paulo, Sao Paulo 05403-000, Brazil; 3 Academic Research Organization, Hospital Israelita Albert Einstein, Sao Paulo 05652-900, Brazil

**Keywords:** familial hypercholesterolemia, atherosclerosis, PCSK9, ANGPTL3

## Abstract

Familial hypercholesterolemia (FH) is associated with an elevated risk of atherosclerosis. The finding of monogenic defects indicates higher atherosclerotic risk in comparison with hypercholesterolemia of other etiologies. However, in heterozygous FH, cardiovascular risk is heterogeneous and depends not only on high cholesterol levels but also on the presence of other biomarkers and genes. The development of atherosclerosis risk scores specific for heterozygous FH and the use of subclinical coronary atherosclerosis imaging help with identifying higher-risk individuals who may benefit from further cholesterol lowering with PCSK9 inhibitors. There is no question about the extreme high risk in homozygous FH, and intensive LDL-cholesterol-lowering therapy must be started as soon as possible. These patients have gained life free of events in comparison with the past, but a high atherosclerosis residual risk persists. Furthermore, there is also the issue of aortic and supra-aortic valve disease development. Newer therapies such as inhibitors of microsomal transfer protein and angiopoietin-like protein 3 have opened the possibility of LDL-cholesterol normalization in homozygous FH and may provide an alternative to lipoprotein apheresis for these patients. Gene-based therapies may provide more definite solutions for lowering high LDL cholesterol and consequent atherosclerosis risk for people with FH.

## 1. New Trends in Genetics, Epidemiology, and Atherosclerotic Cardiovascular Disease Risk in Familial Hypercholesterolemia

The new trends in FH are summarized in Table 1.

### 1.1. Genetics of FH

Familial hypercholesterolemia (FH) is an autosomal disorder caused by genetic variants affecting the removal from plasma of low-density lipoprotein (LDL) [17] and is a cause of early atherosclerotic cardiovascular disease (ASCVD). Most frequently (95% of cases), FH is caused by loss-of-function variants in the low-density lipoprotein receptor (LDLR) gene (*LDLR*) located on chromosome 19. Occasionally the phenotype may be caused by pathogenic variants in apolipoprotein B (*APOB*), gain-of-function variants in proprotein convertase subtilisin/kexin type 9 (*PCSK9*), LDL receptor adaptor protein (*LDLRAP1*) and apolipoprotein E genes (*APOE*) [17,18]. Recently, the *STAP-1* (signal-transducing adaptor family member 1) gene was discarded as a cause of the FH phenotype [19]. Finally, phenocopies of FH may occur due to defects in *ABCG5*/*ABCG8* (sitosterolemia) [20] and *LIPA* (lysosomal acid lipase) [17].

*LDLR* is responsible for codifying the expression of LDL receptors on the extracellular surfaces of many types of cells, most important in hepatocytes, where they function to bind and internalize circulating LDL into cells for onward catabolism [17,18]. Loss-of-function variants in *LDLR* result in the reduced capacity of the cell–surface mechanism to bind and internalize circulating LDL particles and thereby lead to hypercholesterolemia.

In heterozygous FH, a single mutant allele of *LDLR*, *APOB* or *PCSK9* is inherited from either of the parents carrying the genetic variant, while in homozygous FH, two variants are inherited, one from each parent [17,18], thereby following a co-dominant pattern. Consequently, individuals with homozygous FH typically present a more severe phenotype than heterozygous FH [3]. In most situations, the homozygous FH phenotype results from the inheritance of the same defective allele of the same gene from each parent (i.e., true homozygotes, in most situations the LDLR gene). However, it may also occur from the inheritance of two different alleles of the same gene, one from each parent (compound heterozygotes) or from alleles of different genes (double heterozygotes). The homozygous FH phenotype may also occur as the inheritance of one recessive allele of *LDLRAP1* from each parent (autosomal recessive hypercholesterolemia) [17].

The phenotype severity and thus ASCVD risk in FH [2] may also be consequent to additional inheritance of small-effect genes that when aggregated further raise LDL-cholesterol (LDL-C) additionally to variants on the FH canonical genes [2]. These genes can be evaluated based on polygenic scores, and their effects may explain phenotypic variability in individuals from the same family bearing similar variants in the canonical genes. This occurs due to the variable transmission of these small-effect genes [21].

### 1.2. Epidemiology

A recent meta-analysis indicates a global prevalence of 1:313 individuals for heterozygous FH [22]. Homozygous FH is rare disease with a global prevalence estimated in (1:160,000–300,000) in the general population [23]. However, the prevalence of FH varies according to world region as shown in a recent registry from the Arabian Gulf region that showed an estimated heterozygous FH prevalence of 1:112, about 3-fold the estimated prevalence worldwide [22,24]. The latter may have important implications for the occurrence of cases of homozygous FH in Arabia considering its elevated rates of consanguineous marriages.

### 1.3. Heterogeneity in Atherosclerotic Cardiovascular Disease Risk

The pathophysiologic hallmark of FH is the increased build-up of atherosclerosis due to cumulative lifetime exposure to high-circulating LDL-C concentrations [25]. The lifetime cardiovascular events risk for untreated heterozygous FH individuals was estimated as 3.9-fold (88% absolute risk during lifetime) greater than that of non-FH subjects presenting a similar risk profile except for plasma cholesterol concentrations [26]. Khera et al. encountered a 3.7-fold higher risk of coronary artery disease presence in molecularly proven FH individuals in comparison with people with severe hypercholesterolemia (LDL-C ≥ 190 mg/dL), where FH-causing variants in the canonical genes were not encountered [1].

A recent multinational registry with 61,612 individuals with the diagnosis of heterozygous FH from 56 countries (42,167 adults, mean age 46.2 years, 53.6% women) indicates an ASCVD frequency of 17.4% (2.1% for stroke and 5.2% for peripheral artery disease) [27]. For homozygous FH, a similar registry comprising 751 patients with the phenotype (75% with proven molecular diagnosis) from 38 countries (median age of diagnosis 12.0 years, 52% females) indicates a prevalence of clinical ASCVD or aortic stenosis of 9% at diagnosis [28]. Another study, this one from 8 Iberoamerican countries, comprised 134 individuals with the homozygous FH phenotype, 71 adults (mean age 39.3 years, 62% females) and 63 children (mean age 8.8 years, 51.2% females), 96% of them with confirmed molecular diagnosis [29]. The prevalence of clinical or subclinical ASCVD and aortic or supra-aortic valve diseases was 48% and 67%, respectively, in children and adults. Indeed, the advent of statin and lipoprotein apheresis therapies changed the natural history of homozygous FH with reductions in both coronary heart and supra-aortic valve diseases but with a persistence of calcified aortic valve stenosis [9,10]. Whether early diagnosis and aggressive LDL-C lowering will modify the course of aortic valve disease remains to be determined.

Despite a higher ASCVD risk in comparison with the one encountered in the general population, the latter varies significantly among individuals with heterozygous FH. This risk depends not only on type of molecular defect on the FH canonical genes and consequent LDL-C concentrations but also on the presence of risk biomarkers such as older age, male sex, smoking, low HDL-C, obesity, late onset of lipid-lowering therapy, higher lipoprotein(a) [Lp(a)] concentrations and presence of subclinical coronary atherosclerosis [3,4,30].

Even considering the robust effects of the autosomal dominant molecular defects on LDL-C and the ensuing ASCVD risk other genes may influence on the onset of the latter in people affected by FH [2,5]. In addition to genes that modify LDL-C concentrations [2], other variants that predispose to atherosclerosis development may influence coronary heart disease variability. Fahed et al. [5] elegantly showed that the risk of the latter varied from 18% to 76% at the age of 75 years depending on the percentile of a polygenic atherosclerosis risk score in individuals with monogenic FH-causing defects.

Currently, there is consensus that adults with FH should receive the highest-tolerated dose of statins with the aim of reducing LDL-C by at least 50% [3,31,32]. Ezetimibe therapy can also add 15–20% further LDL-C reduction with safety and low cost. PCSK9 inhibitors, e.g., the monoclonal antibodies alirocumab and evolocumab and the recently approved small-interference RNA inclisiran may add up to 40–60% average reduction in LDL-C in adults with heterozygous FH [33,34,35]. In pediatric heterozygous FH patients, evolocumab was approved after the age of 10 years and provides an additional 35–44% LDL-C reduction on top of that from usual care [36,37].

Consensus documents are unanimous in recommending PCSK9 inhibitors for heterozygous FH patients for secondary prevention [3,31,32] since most will persist with elevated LDL-C concentrations despite statin and ezetimibe therapy [38]. However, the reduced access to PCSK9 inhibitors when reimbursement is concerned precludes a more widespread use of these safe and efficacious therapies. There is evidence from prospective studies that risk stratification with clinical risk scores such as the SAFEHEART risk equation [7] and the Familial Hypercholesterolemia Risk Score [8] may help with gauging ASCVD risk in heterozygous FH. Furthermore, coronary artery calcification detected on cardiac computed tomography [6], a marker of atherosclerotic plaque burden, alone or when added to the latter [4,39], may help discriminate primary prevention heterozygous FH patients according to level of risk. In the 45% of heterozygous FH patients where CAC is absent (calcium score of zero) [40], there is an indication [4,6] that statins and ezetimibe reduced ASCVD risk [14] and no further therapies may be necessary in median 2.7- to 3.7-year follow-ups [41]; however this needs further confirmation from longer-term studies.

**Figure 1:**
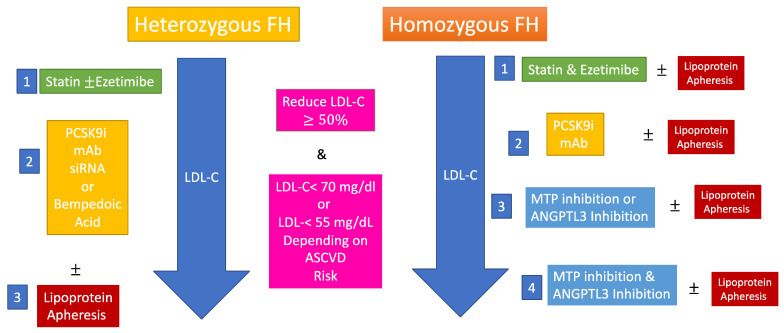
Treatment algorithms for heterozygous and homozygous familial hypercholesterolemia. Here are shown treatment algorithms for both heterozygous and homozygous forms of familial hypercholesterolemia. LDL-C reductions are recommended according to guidelines and consensus papers [3,32]. All adult patients should have at least a ≥50% reduction in LDL-C. Further goals of <70 mg/dL or 55 mg/dL are based on ASCVD risk (high or very high). Recommendations are based on approved drugs, especially for homozygous FH. For homozygous FH, lipid apheresis can be started at all algorithm levels. Drug choices should be based on regulatory issues, country approval and availability. In homozygous FH, PCSK9 inhibitors should be suspended if they do not provide adequate LDL-C and one should move from step 2 to 3. mAB-monoclonal antibody; siRNA-small interfering RNA.

For homozygous FH, there is no discussion of the need to use all available therapies to reduce LDL-C such as statins, ezetimibe, and PCSK9 inhibitors if patients are responsive [33,42]. Indeed, lipoprotein apheresis has been instrumental in LDL-C reduction in people with both severe heterozygous and homozygous FH [43]. There is evidence from observational studies that lipoprotein apheresis not only reduces atherosclerosis progression but also increases ASCVD event-free survival [44]. Indeed, the procedure can be used very early in the therapy algorithm of homozygous FH (Figure 1). Unfortunately, lipoprotein apheresis is not widely available, it has high monetary cost and it is not reimbursed in most countries. Therefore, robust pharmacological LDL-C-lowering therapies that act independently of LDLR such as the MTP (microsomal triglyceride transfer protein) inhibitor lomitapide [15,45] or the recently approved anti-ANGPTL3 (angiopoietin like 3 protein) monoclonal antibody evinacumab [16] are necessary [3]. These therapies may indeed reduce LDL-C concentrations to values seen in the general population [16]. However, there are severe cost issues with either apheresis or the new pharmacological therapies, and therefore access to them is low in most countries, as shown in the two recent published homozygous FH registries [28,29].

Given that the risk and severity of ASCVD in FH patients vary depending on the type of genetic defects [17,18] as well as other risk biomarkers [18], and considering the development of novel therapies, the treatment of FH patients is changing from generalized to individualized (tailored) approaches. The following part of the present review aims to evaluate the trends in the of therapies for FH.

### 1.4. Impact of Statin Therapy and Ezetimibe on LDL-C and ASCVD Risk in FH

Statin therapy is a lifelong preventive treatment of choice for individuals with FH [46]. Statins reduce LDL-C by diminishing hepatic cholesterol synthesis acting on the 3-hydroxy-3-methylglutaryl coenzyme-A (HMG-CoA)-reductase. That leads to the upregulation of hepatic LDLR expression and the increased uptake of circulating LDL particles with consequent higher biliary cholesterol excretion in the feces [47]. Reductions in cholesterol synthesis also result in less production of VLDL (very-low-density lipoprotein), a precursor of LDL. Overall, statins reduce the hepatic output of cholesterol to peripheral arteries, hence decreasing cholesterol plaque buildup assessed by a surrogate progression of carotid intima–media thickening and the risk of ASCVD events and mortality [12,46,48].

Several observational studies have indicated that statin therapy protects against ASCVD in people with FH [10,11,12,13]. In a cohort study, Versmissen et al. [12] enrolled 2146 patients with a confirmed genetic diagnosis of FH without previous coronary heart disease (CHD) manifestation from 27 outpatient lipid clinics in the Netherlands. The cohort was started on statin therapy (with lower doses of simvastatin, 33 mg/day or atorvastatin, 49 mg/day) and monitored for risk of CHD during a mean follow-up period of 8.5 years. At the end of the follow-up, statin therapy had reduced LDL-C by an average 44% in comparison with the baseline and the risk of myocardial infarction by 76% (hazard ratio, 0.24 [95% confidence interval, 0.18 to 0.30]) to a level comparable with that of the age-matched general population (hazard ratio, 1.44 95% confidence interval 0.80 to 2.60) [12]. However, findings from the study by Versmissen et al. [12] should be interpreted with caution considering its observational nature, lack of a randomized control group and wide confidence intervals when the risk was compared with the one from the non-FH population.

Acknowledging the differential ASCVD risk between heterozygous and homozygous FH subpopulations, three studies have evaluated the impact of statin therapy in these groups individually [11,13,49]. An observational multicenter study by Besseling et al. [13] evaluated the risk of CHD in individuals with heterozygous FH that had been diagnosed and followed in the Netherlands cascade screening program. A total of 1559 patients free of coronary artery disease (CAD) at baseline were started on 40 mg/day of either simvastatin (23.1%) and atorvastatin (22.8%) (*n* = 1041) or no treatment (*n* = 518). There were, respectively, 89 and 17 CAD and mortality events during 11,674 person-years of follow-up in the statin group. These values were significantly lower than the 22 and 9 events, respectively, during the 4892 person-years of follow-up in the non-statin group. Overall, the rates of CAD events were 5.3 vs. 8.8 per 1000 person-years in those receiving statins or not; *p* < 0.001. After adjustment for confounders, the rates of CAD and all-cause mortality were reduced by 44% with a hazard ratio of 0.56 (95% confidence interval: 0.33 to 0.96).

A 20-year prospective observational study [49] comprising 214 Dutch pediatric FH patients (98% with confirmed molecular diagnosis) emphasized the need for early cholesterol-lowering therapy in heterozygous FH. A 32% reduction in LDL-C with statins (mostly pravastatin) was associated with the normalization of carotid intima–media thickness progression when compared with nonaffected siblings. Most important, there were reductions in ASCVD and cardiovascular mortality when compared with parents who had not been treated in the past. At the age of 39 years, cardiovascular events and mortality were, respectively, 1% and 26% and 0% and 7%, respectively, in children who became adults and in their untreated parents. Therapy was safe and well tolerated. The results of course should be interpreted considering the limitations inherent in an observational study.

Raal et al. [11] evaluated the effect of statin therapy on CVD morbidity and mortality in a cohort of 149 homozygous FH patients (81 females) from two specialized lipid clinics in South Africa. The hazard ratios of the benefit from lipid-lowering therapy, mostly with statins, were 0.49 (95% confidence interval: 0.22–1.07; *p* = 0.07) and 0.34 (95% CI: 0.14–0.86; *p* = 0.02) for major adverse cardiovascular disease events and death, respectively. Surprisingly, results were seen despite a mean relative reduction in LDL-C of only 26.4% (from 620 ± 152 to 456 ± 132 mg/dL).

Ezetimibe is prescribed as a second-line therapy for LDL-C lowering in FH patients who persist with inadequate LDL-C concentrations [50]. It is a cholesterol-absorption inhibitor that acts at the “brush border” of the inner wall of the small intestines. Ezetimibe binds the sterol transporter protein, called Niemann-Pick C1 like 1 (NPC1L1) protein, hence reducing intestinal cholesterol absorption and increasing its fecal excretion [51]. Ezetimibe usually adds 10–15% additional LDL-C reduction to isolated statin therapy, and in both heterozygous and homozygous FH patients, a significant further absolute reduction in LDL-C levels is achieved by combination therapy [52,53]. Indeed, even considering the lower impact on LDL-C reduction when compared with PCSK9 inhibitors, there is a strong recommendation to start with the highest-tolerated statin dose and ezetimibe combination rather than statin therapy alone in people with FH [54]. However, particularly in those with FH and previous ASCVD or in those with high subclinical atherosclerosis burden, there is the unmet need of still-elevated LDL-C [14,38], and these patients will often require a third-line pharmacological intervention such as PCSK9 inhibitors to achieve the target LDL-C goals [55,56]. PCSK9 inhibitors are among the current targeted molecular therapies for FH, which are discussed in detail in the subsequent sections.

## 2. New Trends in Therapies for FH

Several new targeted therapies were developed, and some are being tested to achieve the LDL-C goals for high-/very-high-risk FH patients as recommended by the ESC/EAS 2016–2019 guidelines [50]. The mechanism of action and impact on LDL-C plasma concentrations for both heterozygous and homozygous FH are shown in Table 2. The advantage of targeted therapies is that they provide clinicians with the power to practice personalized or precision medicine with the aim of achieving better risk/benefit and cost/effectiveness of therapies. This is important considering the elevated costs of monoclonal antibodies and RNA-targeted therapies.

### 2.1. PCSK9 Inhibitors

PCSK9 inhibitors are a new class of cholesterol-lowering drugs currently used as a third-line treatment for FH or for statin-intolerant or very-high-ASCVD-risk patients. PCSK9 is an enzyme produced mainly in the liver that is secreted into the plasma and plays a critical role in LDL catabolism. LDL normally clears from peripheral blood as a complex with the LDLR that enters the hepatocyte [63]. PCSK9 binds the LDLR at the hepatocyte surface, reducing its recycling from cytoplasm to cell membrane and consequently diminishing LDL clearance [64]. A previous animal study demonstrated that mice overexpressing PCSK9 protein have decreased LDLR function and elevated plasma LDL-C, while PCSK9 knockout mice have increased LDLR activity and lower plasma LDL-C levels [65]. Studies in humans showed that gain- and loss-of-function variants in PCSK9 were associated with an FH phenotype [66] and lower LDL-C concentrations and ASCVD risk [67], respectively.

These findings formed the basis for the development of PCSK9 inhibitors, whose mechanisms, as the name suggests inhibits the activity of PCSK9 proteins via different mechanisms. Three different subclasses of PCSK9 inhibitors are discussed.

Human monoclonal antibodies against PCSK9 (PCSK9-mAb) primarily include alirocumab and evolocumab [33,42,68]. The primary mechanism of action of PCSK9-mAb is via their binding activity on PCSK9 in the plasma, thereby blocking PCSK9 from binding the LDLR. The latter means that more receptors are available for the binding of ApoB100-LDL complex for the onward clearance of circulating LDL particles [47,69].

Alirocumab and evolocumab are indicated for homozygous and heterozygous FH patients who persist with elevated LDL-C despite the use of statins and ezetimibe therapies. They are administered subcutaneously in bimonthly doses of 75–150 mg for alirocumab or 140 mg for evolocumab or 300 mg and 420 mg, respectively, for alirocumab and evolocumab once monthly. A recent open-label, single-arm multicenter study by Santos et al. (TAUSSIG) [38] evaluated the safety and efficacy of evolocumab in 300 patients aged ≥12 years with HoFH (*n* = 106) and severe HeFH (*n* = 194) who at the time of enrolment were on stable lipid-lowering therapy. Patients were started on evolocumab (420 mg monthly and later to 420 mg bimonthly as needed) or 420 mg bimonthly if on lipoprotein apheresis. At 12 weeks of evolocumab treatment, LDL-C decreased by 59.8 mg/dL (21.2%) and 104.4 mg/dL (54.9%) in patients with HoFH and HeFH, respectively; effects were sustained during a median follow-up of 4.1 years. A total of 26% of patients on active apheresis (severe heterozygous FH only) had their blood-filtering therapy discontinued to LDL-C control, and the overall rate of CVD events was only 2.7%, suggesting cardiovascular benefit of the drug in comparison with historical controls. Adverse reactions occurred in 89.3% of patients, which included nasopharyngitis, influenza, upper respiratory tract infection and headache [38]. The latter, however, did not lead to drug discontinuation. In the same study, Raal et al. [7] demonstrated that in homozygous FH, the presence of *LDLR* null variants was associated with a lower or absent reduction in LDL-C with evolocumab in comparison with those with defective variants.

Blom et al. evaluated the effects of alirocumab in homozygous FH [8]. In a randomized, double-blind, placebo-controlled, parallel-group study, 69 patients on high-intensity lipid-lowering therapy including statins, ezetimibe, lomitapide and apheresis were enrolled. Patients were randomized to receive 150 mg alirocumab treatment every 2 weeks (*n* = 46; baseline LDL-C = 295 mg/dL) or placebo (*n* = 23; baseline LDL-C = 259 mg/dL) for a duration of 12 weeks. Alirocumab significantly decreased LDL-C by 26.9% compared with 8.6% for placebo (*p* < 0.0001). Similar to evolocumab [38], alirocumab was generally well tolerated, with a safety profile comparable with that of placebo [8]. Both studies show that PCSK9-mAb may be useful for LDL-C reduction in either severe heterozygous or homozygous FH, although the efficacy in the latter is much less pronounced.

Evolocumab was approved for pediatric patients (older than 10 years) with HeFH based on results from the HAUSER trial [36]. In HAUSER, evolocumab (420 mg once a month) was administered in a randomized 2:1 double-blind fashion to 157 pediatric patients (mean age 13.7 years) who persisted with LDL-C > 135 mg/dL despite usual statin and/or ezetimibe therapy (mean baseline LDL-C 185 ± 45 mg/dL). After 24 weeks, there was a mean 38.3% reduction (−44.5% vs. −6.2%) in LDL-C versus placebo. Adverse events were similar in comparison with placebo, with nasopharyngitis and headache being the most frequent. Recently Santos et al. [37] have published the long-term open label follow-up of HAUSER. In that study, 150 patients received evolocumab and completed the open-label extension with a median follow-up of 80.3 weeks. The main study objective was safety and tolerability; treatment-associated adverse events occurred in 70% of study participants and were similar to the ones occurring in the randomized double-blind phase. No event led to treatment discontinuation, and no patient developed anti-drug antibodies. There were no adverse events related to growth, sexual maturation, neurocognitive function, glucose homeostasis, steroid hormones or liposoluble-vitamin blood concentrations. At week 80, the mean percentage change from baseline in LDL cholesterol was −35.3% (standard deviation 28.0). The study clearly shows that evolocumab can add LDL-C reduction to usual therapy in heterozygous FH, is safe and is well tolerated.

Small-interfering RNA (siRNA) technology is a novel approach to PCSK9 inhibition [60]. The siRNA technology deploys a small double-stranded RNA molecule of 19–23 nucleotides in size to induce the silencing of the target gene. The siRNA inclisiran is a novel PCSK9 inhibitor for the treatment of heterozygous FH and common hypercholesterolemia [60]. Inclisiran blocks the translation of PCSK9 messenger RNA, leading to its degradation by the RNA-induced silencing complex (RISC) andthereby decreasing the concentrations of intrahepatic and plasma PCSK9 [35,60]. Compared with PCSK9-mAbs, inclisiran has a more convenient dose regimen of 300 mg twice-yearly injections and could be useful for enhancing patient compliance. In a recent phase 3, double-blind trial by Raal et al. [35], 482 adult heterozygous FH patients were randomized to receive 300 mg of inclisiran (*n* = 241) or matching placebo (*n* = 241) and followed up for 540 days. The mean reduction in the LDL-C level from day 90 to day 540 was 38.1% in the inclisiran group, while there was an increase of 6.2% in placebo (*p* < 0.001), a −44.3% difference. The most frequent adverse events not differing from placebo were nasopharyngitis, influenza, upper respiratory tract infection and back pain. In a pilot study, Hovingh et al. [70] tested the feasibility of PCSK9 suppression with inclisiran in four homozygous FH patients. LDL-C changes varied from +3% to −37% in 180 days, and PCSK9 plasma levels were reduced by −48.7% to −83.6% at day 90 and by −40.2% to −80.5% at day 180. This study paved the way for ORION 5 (NCT03851705) with a greater number of homozygous FH patients. However, results are not yet published.

At any rate, the infrequent dosing regimen and acceptable safety profile of inclisiran make it a suitable alternative to PCSK9-mAbs [35].

Oral PCSK9 inhibitors are being developed as an alternative to the subcutaneous PCSK9-mAbs and inclisiran. This presentation may be especially suitable for FH patients who cannot comply with subcutaneous injections of PCSK9-mAbs and inclisiran. MK-0616 is an orally bioavailable PCSK9 inhibitor and preliminary results from an ongoing phase I clinical trial by Johns et al. [71] were presented at the 2021 Scientific Sessions of the American Heart Association. The study involved 60 healthy male volunteers and has demonstrated that MK-0616 (10–300 mg) was well-tolerated with no adverse effects. In the second phase of the study, involving 40 hypercholesterolemic patients (male and female), MK-0616 lowered baseline LDL-C levels by 65% after 14 days of treatment. Further data are, however, necessary.

Gennemark et al. [72] developed a chemically modified PCSK9 antisense oligonucleotide (ASO) for oral delivery. Preliminary results showed that the subcutaneous injection of 90 mg ASO reduced PCSK9 by >90% in patients with elevated LDL-C levels with a predicted 80% steady state with a 25 mg monthly maintenance dose [72]. When ASO was co-formulated with sodium caprate (a permeation enhancer) in an oral tablet form and administered to dogs, it resulted in 7% hepatic bioavailability, which was 5 times greater than that of plasma. Using prediction models, 15 mg/day of oral ASO should suppress PCSK9 in peripheral blood by 80% steady state and therefore be viable for oral formulation [72].

PCSK9 inhibitors are frequently prescribed as third-line treatment in patients who could not respond well or tolerate conventional lipid-lowering therapies. They are ideal for those with heterozygous FH [73]. However, despite the use of high-dose statins and ezetimibe in combination with PCSK9 inhibitors, many patients with homozygous FH fail to achieve optimal reductions of LDL-C levels [74]. Thus, more treatment strategies are still needed.

### 2.2. Bempedoic Acid

Bempedoic acid (BA) is an oral inhibitor of cholesterol biosynthesis approved for cholesterol reduction [58]. Its mechanism of action involves the inhibition of the adenosine triphosphate citrate lyase (ACL), which acts upstream of HMG-CoA-reductase in the cholesterol biosynthesis pathway. A recent randomized controlled trial enrolled 2230 patients with ASCVD, heterozygous FH or both, all on the maximum tolerated dose of statin monotherapy (mean baseline LDL-C = 103.2 ± 29.4 mg/dL) to receive either BA treatment (*n* = 1488) or placebo (*n* = 742). During the 52 weeks of treatment, the incidence of adverse effects was comparable between the two groups. At week 12, the BA group exhibited significant LDL-C reduction from baseline (16.5%). At 52 weeks, BA did not result in higher incident of adverse effects and LDL-C lowering effects were maintained [75]. Similar results, a 21% reduction in LDL-C level compared with placebo, were reported after 12 weeks in another randomized study that evaluated BA in patients with hypercholesterolemia and statin intolerance [76]. These findings, in general, indicate that BA is efficacious and safe for lowering LDL-C in patients with hyperlipidemia including heterozygous FH patients.

### 2.3. Angiopoietin-like 3 Protein (ANGPTL3) Inhibitors

Angiopoietin-like 3 protein (ANGPTL3) is an endogenous inhibitor of lipoprotein and endothelial lipases. Loss-of-function variants of the ANGPTL3 gene are associated with lower serum cholesterol and triglyceride levels and a lower ASCVD risk [77]. Evinacumab, a human monoclonal antibody for ANGPTL3 inhibition (ANGPTL3-mAbs), was approved for the treatment of adults and pediatric patients older than 12 years with homozygous FH [16,62]. Raal et al. [16] showed in a randomized double blind study that 15 mg/kg infusions of evinacumab every 4 weeks reduced LDL-c by 49% at week 24 in comparison with placebo in homozygous FH patients (*n* = 65, baseline LDL-C of 255 mg/dL) undergoing maximal lipid-lowering therapies (statins, ezetimibe, PCSK9 inhibitors, lomitapide and/or lipoprotein apheresis). Of importance, and different, from PCSK9 inhibitors, evinacumab provided robust LDL-C reduction (−43.4%) even in patients with *LDLR* null variants. Adverse events did not differ from placebo. Animal models suggest that evinacumab increases the removal of VLDL and IDL particles, precursors of LDL, by a non-LDLR related pathway [61].

In another double-blind, placebo-controlled, phase 2 trial, Rosenson et al. [46] enrolled 272 patients with and without heterozygous FH and with evidence of refractory hypercholesterolemia with atherosclerosis (LDL-C levels ≥ 70 mg/dL) or without atherosclerosis (LDL-c levels ≥ 100 mg/dL). Patients were randomly assigned to receive evinacumab at various dosing regimens (either subcutaneous or intravenous) or a placebo. After 16 weeks, evinacumab lowered LDL-C by 38.5–56% according to dose regimen compared with placebo, with low incidence of adverse effects (3–16%) across trial groups. Evinacumab is not yet approved for refractory heterozygous FH. Despite favorable results with monoclonal antibodies against ANGPTL3, recently, the development of an ASO against that protein, vupanorsen, was interrupted due to adverse liver events [78]. This clearly demonstrates that despite similar targets, different technologies vary when safety is concerned, and more clinical studies are warranted.

### 2.4. MTP Inhibitors

MTP is an enzyme essential for the assembly of VLDL in hepatocytes and chylomicrons in enterocytes. The inhibition of MTP blocks VLDL assembly and reduces LDL-C levels [47]. Lomitapide is an MTP inhibitor used as a lipid-lowering agent approved for the treatment of homozygous FH patients [15,59]. A previous single-arm, open-label, phase 3 multicenter study by Cuchel et al. [59] enrolled 29 homozygous FH patients to receive lomitapide in doses ranging from 5 to 60 mg/day depending on safety and tolerability. Lomitapide achieved a 50% reduction in baseline LDL-C levels at 26 weeks and maintained steady states of 44% and 38% at weeks 56 and 78, respectively. However, the drug produced gastrointestinal adverse effects and liver steatosis, though this did not result in discontinuation.

Two studies [15,45] have provided long-term efficacy and safety data on lomitapide in patients with homozygous FH treated up to 5.9 years. Blom et al. [15], in an extension of the original study by Cuchel et al. [59], showed in 17 patients followed up for 5.1 years that lomitapide in a 40 mg dose reduced LDL-C by 45% with hepatic safety. The most important reported adverse events were diarrhea, nausea, dyspepsia and vomiting. Underberg et al. [45] showed in the LOWER-registry that the median lomitapide dose of 10 mg provided a sustained 33% reduction in LDL-C after 5.9 years. In those who remained on lomitapide therapy until the end of follow-up, LDL-C reduction was 45%, with 65.4% and 41.1% achieving an LDL-C < 100 mg/dL or <70 mg/dL, respectively. Treatment-related adverse events occurred in 54.6%, and 23.2% of patients, who discontinued the therapy due to that. Gastrointestinal and hepatic events occurred, respectively, in 13.5% and 15.1%. Overall, the studies reported consistent results demonstrating that lomitapide, when used in combination with other lipid-lowering therapies is effective in lowering LDL-C levels with an acceptable tolerability and safety profile [15,45].

Ben-Omran et al. [79] evaluated the effects of lomitapide (mean dose 24.5 ± 4.3 mg/day; during 20.0 ± 2.9 months) in a case series of 11 pediatric homozygous FH patients, mean age 11.6 ± 1.1 years and 64% males, undergoing statin and or ezetimibe therapy. LDL-C was reduced by 58.4 ± 6.8% from a baseline of 419 ± 74.6 mg/dL. The most frequent adverse events were nausea, vomiting and diarrhea but were well tolerated. A phase III study (NCT04681170) is testing the efficacy and safety of lomitapide in pediatric homozygous FH patients aged 5–17 years old with a duration of up to 80 weeks.

### 2.5. Gene Therapies

People affected by FH are ideal candidates for gene therapy, which is potentially the most definitive treatment for life. Possible gene therapies include CRISPR/Cas9 for heterozygous FH and viral vectors for homozygotes.

The Clustered Regularly Interspaced Short Palindromic Repeat (CRISPR)-Associated System 9 (Cas9) or simply CRISPR/Cas9 is the most promising genome editing tool for model systems including animal zygotes and human cells. CRISPR/Cas9 has useful applications in genetic research and is a promising tool for clinical applications in treating genetic disorders [80].

A recent in vivo animal study used a recombinant adeno-associated virus (AAV) vector carrying CRISPR/Cas9 gene editor (AAV-CRISPR/Cas9) and targeting an LDLR mutant mice model [81]. The mutant mice with loss of LDLR function exhibited severe atherosclerotic phenotypes when fed a high-fat diet. The AAV-CRISPR/Cas9-mediated gene editing partially corrected the point mutation in the LDLR gene expressed in hepatocytes and restored partial LDLR protein expression. The treatment significantly decreased total cholesterol, triglycerides, and LDL-c in the serum and, consequently, decreased the build-up of atherosclerotic plaques in the aorta. This finding shows that CRISPR/Cas9 is promising for the treatment of heterozygous FH.

A recombinant adeno-associated virus (AAV) vector carrying an *LDLR* transgene has been recently unveiled and is currently at phase 1/2a testing phase [82]. In animal studies, an LDLR-deficient mouse model (Ldlr^−/−^, Apobec1^−/−^ or double knockout—DKO) treated with AAV carrying an LDLR transgene at vectors doses as low as 3 × 10^11^ exhibited enhanced transgene expression and decreased serum LDL-C levels [83]. Findings from DKO mice indicate the potential of an AAV vector carrying an LDLR transgene to be used for the treatment of high-risk homozygous FH patients.

## 3. Conclusions

FH is a disease associated with an elevated risk of ASCVD that needs to be early diagnosed and adequately treated. The diagnosis of FH has implications not only for the index case but also for the relatives who need to be identified by cascade screening [17]. The molecular diagnosis is important since the presence of monogenic defects implies a higher ASCVD risk in comparison with hypercholesterolemia of other etiologies [1]. Despite the elevated atherosclerosis [3,30] risk in heterozygous FH, the latter is heterogeneous and depends not only on cholesterol levels but also on the presence or not of other genes and biomarkers. The development of ASCVD risk scores specific for FH and the use of coronary subclinical atherosclerosis imaging may help the institution of targeted further LDL-C-lowering therapies on top of statins and ezetimibe in heterozygous FH [14]. This is important considering the low worldwide access to novel robust LDL-C-lowering therapies such as the PCSK9 inhibitors.

There is no question about the extreme high risk to people with homozygous FH, and intensive LDL-C-lowering therapy must be started as soon as possible. These patients have gained life free of events in comparison with the past [10,11], but residual risk is still extremely elevated, and there is also the issue of aortic valve disease development [9]. The onset of MTP inhibitors [45] and monoclonal antibodies against ANGPTL3 [16] associated or not with lipoprotein apheresis and PCSK9 inhibitors has opened the possibility of the normalization of LDL-C in homozygous FH; these may even provide one alternative to the latter. Figure 1 shows a suggested therapy algorithm for both heterozygous and homozygous FH forms with available approved therapies. The aim is to reduce LDL-C at least 50% and attain the recommended LDL-C goals according to ASCVD risk [3,32], a barrier that with the newer therapies finally became possible to be trespassed. Certainly, there is still the important unmet need of access to these therapies that remains a barrier in most low- to middle-income countries [27,28,29]. Gene-based therapies [80] may deliver more definite solutions to LDL-C and consequent ASCVD risk reduction for people with FH.

## Figures and Tables

**Table 1:** Current facts and new trends in familial hypercholesterolemia.

**Genetics**	1-Monogenic defects implicated in higher atherosclerotic risk in comparison with hypercholesterolemia of other etiologies [1]. 2-LDL-C concentrations depend not only on defects on canonical genes but also on polygenic effects [2].
**Atherosclerosis risk**	1-Risk in heterozygous FH is heterogenous and depends not only on LDL-C but also other biomarkers, genes and subclinical atherosclerosis [3,4,5,6]. 2-Specific FH risk scores [7,8] and coronary atherosclerosis imaging ae useful in risk stratification [4,6]. 3-Risk of homozygous FH is very high, but therapies (drugs and apheresis) have changed the natural history of disease, and with reduction in CHD but persistence of aortic valve disease [9,10,11].
**Therapies**	1-Statin therapy reduces ASCVD risk in FH [12,13] 2-PCSK9 inhibitors have changed the way we treat heterozygous FH but should be used in those at highest risk when not available for all [14]. 3-Combination of statins, ezetimibe, PCSK9 inhibitors, MTP inhibitors and anti ANGPTL3 antibodies may normalize LDL-C in homozygous FH [15,16]. 4-Genetic therapies are being developed for FH and may bring more definitive LDL-C lowering.

**Table 2:** Mechanism of action and efficacy of approved therapies to treat familial hypercholesterolemia.

	Compound	Target	Mechanism of Action	Efficacy in Heterozygous FH (LDL-C Reduction)	Efficacy in Homozygous FH (LDL-C Reduction)
Statins	Small molecule	HMG-CoA-reductase	Reduces cholesterol synthesis and VLDL production. Increases hepatic LDLR expression [47].	30–50% [57].	10–25% [53].
Ezetimibe	Small molecule	NPC1L1	Reduces intestinal cholesterol absorption and increases hepatic LDLR expression [47].	10–15% [57].	10–15% [53].
Bempedoic acid	Small molecule	ACL	Reduces cholesterol synthesis and VLDL production. Increases LDLR expression [58].	16.5% in a pooled group of FH and other hypercholesterolemia patients [58].	N.A.
Lomitapide *	Small molecule	MTP	Reduces VLDL synthesis [47].	N.A.	33–50% depending on the dose [15,45,59].
Alirocumab &Evolocumab	Monoclonal antibody	Circulating PCSK9	Reduces LDLR degradation [47].	50–60% [3,33] adults and 35–38% pediatric patients for evolocumab [36,37].	20–34% (depends on *LDLR* variant 0–50%) [33,42].
Inclisiran	Small-interfering RNAs	Hepatic PCSK9 synthesis	Reduces LDLR degradation [60].	44.3% reduction [35].	Study ongoing.
Evinacumab *	Monoclonal antibody	Circulating ANGPTL3	Possibly increases the removal of VLDL and IDL particles by LDLR independent pathways [61].	38.5–56% reduction depending on dose regimen and patient [62].	49% [16].
Lipoprotein apheresis	Device	Circulating LDL, Lp(a) and VLDL particles	Reduces pro-atherogenic apoB-100-containing lipoproteins LDL, Lp(a), and VLDL as well as pro-inflammatory biomarkers [43].	60–80% [3].	60–80% [3].

Note: HMG-CoA hydroxy methyl glutaryl Coenzime A; NPC1L1- Niemann-Pick C1 Like 1; ACL-Adenosine triphosphate citrate lyase enzyme (ACL); MTP-Microsomal triglyceride transfer protein; PCSK9-proprotein convertase subtilisin/kexin type 9; ANPTL3-Angiopoietin Like 3 Protein); LDLR-LDL receptor; *LDLR*-LDL receptor gene; Lp(a)-lipoprotein(a); * approved only for homozygous FH.

## Data Availability

Not applicable.
